# Case Report: Four-year durable remission, cerebral oligoprogression, and femoral head necrosis following severe recurrent immune checkpoint inhibitor-related pneumonitis in a patient with stage IV lung adenocarcinoma

**DOI:** 10.3389/fphar.2026.1932295

**Published:** 2026-10-15

**Authors:** Yanjun Xue, Aizhen Xu, Xin Tong, Xinda Yin, Lei Wei

**Affiliations:** 1 Department of Respiratory and Critical Care Medicine, Zibo Central Hospital, Zibo, Shandong, China; 2 Department of Radiation Oncology, Zibo Central Hospital, Zibo, Shandong, China

**Keywords:** cerebral oligoprogression, corticosteroids, femoral head necrosis, immune checkpoint inhibitor-related pneumonitis, lung adenocarcinoma, pirfenidone, sintilimab

## Abstract

Severe immune checkpoint inhibitor-related pneumonitis (CIP) typically leads to the permanent discontinuation of immunotherapy; however, its long-term oncological outcomes and late drug toxicity remain poorly defined. This report describes a patient with stage IV lung adenocarcinoma who, following recurrent grade 3 CIP, achieved durable systemic remission for more than 4 years and displayed a distinctive pattern of cerebral oligoprogression. A 66-year-old male patient with stage IV lung adenocarcinoma achieved a partial response (PR) after four cycles of sintilimab combined with chemotherapy. Three weeks after the last dose, he developed grade 3 CIP complicated by acute pulmonary embolism. Recurrence occurred twice because of overly rapid corticosteroid tapering and was ultimately controlled through an ultralow tapering regimen (50 mg/day prednisone initially, reduced by 5 mg per week) combined with pirfenidone for antifibrotic therapy. Following the resolution of CIP, no further systemic antitumor therapy was administered. During 48 months of follow-up, the patient maintained complete remission (CR) of extracranial metastases; the sole site of progression was a solitary cerebellar lesion, which exhibited three episodes of oligoprogression, each of which was successfully managed with either radiotherapy or bevacizumab-based therapy. At 34 months after the onset of CIP, the patient developed bilateral osteonecrosis of the femoral head (ONFH), with right-sided slight collapse (ARCO stage III) and left-sided stage I. This case suggests a potential synergistic toxic mechanism between cumulative corticosteroid exposure (approximately 7,300 mg prednisone equivalents) and VEGF inhibition-mediated microvascular injury. This case also suggests that ultralow corticosteroid tapering combined with antifibrotic therapy can effectively manage refractory CIP; the dissociation between durable peripheral immunological memory and isolated central nervous system (CNS) progression supports the hypothesis of CNS immune privilege. Finally, clinicians must remain vigilant about long-term musculoskeletal toxicity in long-term survivors of ICI therapy.

## 1 Introduction

Immune checkpoint inhibitors (ICIs) have significantly reshaped the treatment landscape for advanced non-small cell lung cancer; however, CIP, a life-threatening adverse event, remains a major clinical challenge. Severe CIP not only has a high incidence rate but also frequently leads to treatment interruption because of difficult corticosteroid tapering or recurrence (Maule et al., 2026; Chinese Thoracic Society, Chinese Medical Association, Oncology Respiratory Disease Committee of China Anti-Cancer Association, Beijing Cancer Prevention and Treatment Research Association, 2025). Currently, long-term follow-up data concerning the duration of durable immunological memory after ICI discontinuation, the patterns of disease recurrence, and the late-onset toxicities associated with prolonged corticosteroid exposure, such as osteonecrosis, are scarce. For patients who survive severe CIP, the optimal balance between subsequent tumor control and drug toxicity has not yet been established. Herein, we report the 4-year follow-up data of a patient with stage IV lung adenocarcinoma who, following recurrent severe CIP and corticosteroid-related complications, exhibited a distinctive “systemic remission with isolated cerebral oligoprogression” dissociation pattern, aiming to provide evidence for individualized toxicity management and long-term pharmacovigilance. As a single case report, this study cannot establish causality, and its findings have inherent limitations in terms of generalizability. The observations made related to this patient should therefore be interpreted with caution and warrant further validation in larger prospective cohorts.

## 2 Case report

### 2.1 Patient demographics and initial presentation

The patient was a 66-year-old male with a history of hypertension and a 40-pack-year smoking history. In April 2022, he was diagnosed with stage IV left lung adenocarcinoma with cervical lymph node and adrenal metastases. Small-panel next-generation sequencing revealed no actionable driver gene mutations. PD-L1 expression data and TMB data were unavailable because residual tumor tissue was insufficient for PD-L1 immunohistochemistry and because large-panel next-generation sequencing for TMB was not available at our institution at that time. The patient’s Eastern Cooperative Oncology Group performance status (ECOG PS) was 0.

From April to June 2022, the patient received four cycles of first-line cisplatin, pemetrexed, and sintilimab, and the treatment response was assessed as PR.

### 2.2 Onset of CIP and management of CIP recurrence

Approximately 3 weeks after the last dose of sintilimab, the patient was hospitalized because of progressive dyspnea. Chest CT revealed new bilateral ground-glass opacities (Figure 1A). As antibiotic therapy was ineffective, CIP was clinically suspected, and intravenous methylprednisolone at 40 mg/day was started. Both symptoms and imaging findings improved markedly within 7 days (Figure 1B), and corticosteroids were discontinued upon discharge.

**FIGURE 1:**
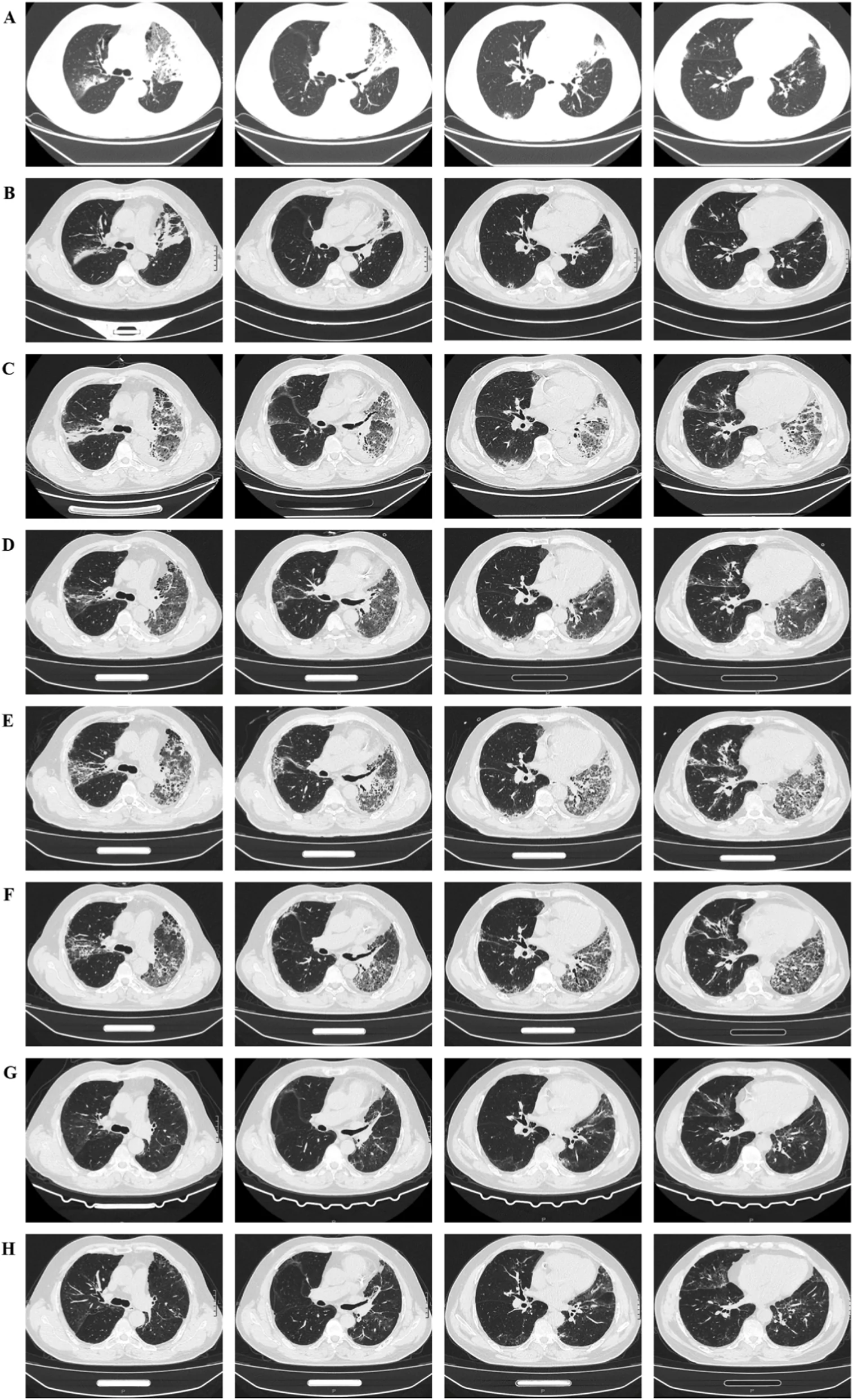
Serial chest CT images taken over the course of checkpoint inhibitor-related pneumonitis (CIP). **(A)** At initial CIP presentation (July 2022, approximately 3 weeks after the last dose of sintilimab), showing bilateral ground-glass opacities. **(B)** After 7 days of initial corticosteroid therapy (July 2022), showing marked improvement. **(C)** At the first CIP recurrence (August 2022), showing radiological progression. **(D)** After dose escalation to 160 mg/day methylprednisolone for 7 days (August 2022), showing improvement. **(E)** At the second CIP recurrence (September 2022), on the sixth day of 30 mg/day prednisone administration, showing radiological progression. **(F)** After re-escalation to methylprednisolone at 160 mg/day, followed by a reduction to 80 mg/day (September 2022), showing continued improvement. **(G)** At 2 months after discharge (November 2022), showing significant resolution of pulmonary inflammation. **(H)** At 5 months after discharge (February 2023), showing complete resolution of interstitial changes.

One month later, the patient developed recurrent dyspnea with radiological progression (Figure 1C). CT pulmonary angiography (CTPA) revealed segmental pulmonary embolism in the right lung (Figure 2), with a plasma D-dimer concentration of 1.11 mg/L FEU. Despite treatment with broad-spectrum antibiotics, methylprednisolone at 40 mg/day, and anticoagulation therapy, the patient’s hypoxemia worsened, with the oxygenation index declining to 200 mmHg. After infection and autoimmune etiologies were excluded, a diagnosis of recurrent grade 3 CIP was confirmed. The methylprednisolone dose was increased to 160 mg/day (2 mg/kg/day); after 9 days of treatment, symptoms and imaging findings improved (Figure 1D). The dose was then reduced to 80 mg/day for 3 days, after which the patient was discharged; after discharge, treatment was switched to oral prednisone 40 mg/day, tapered by 10 mg per week. For anticoagulation therapy, the patient received 100 AXaIU/kg enoxaparin twice daily during hospitalization for 17 days; after discharge, 15 mg rivaroxaban twice daily was given for the first 5 days, followed by 20 mg rivaroxaban once daily as maintenance therapy. After 3 months of anticoagulation therapy, follow-up CTPA revealed complete resolution of the pulmonary thrombi without residual embolism; 20 mg rivaroxaban once daily was continued for another 3 months, resulting in a total anticoagulation duration of 6 months. No bleeding complications occurred during anticoagulation therapy.

**FIGURE 2:**
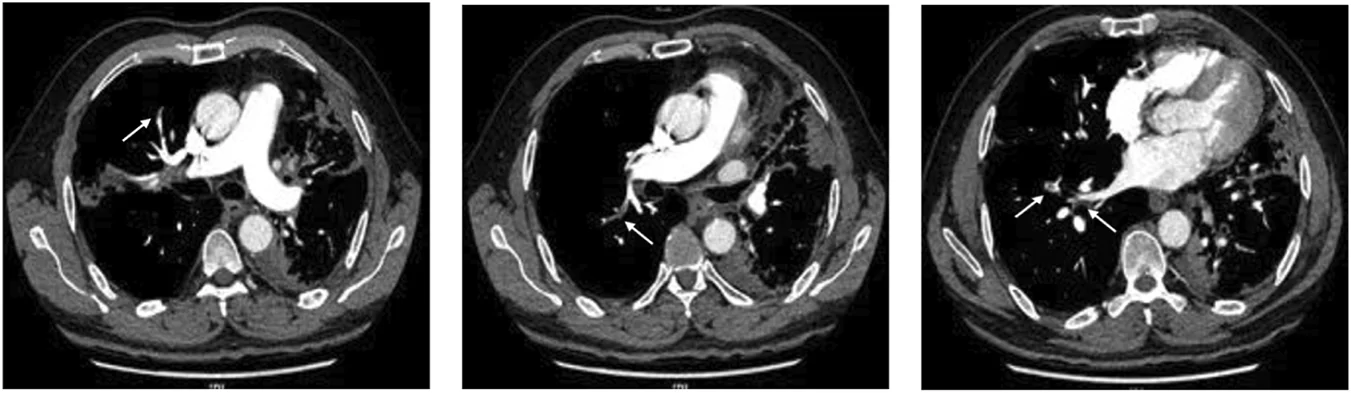
Pulmonary angiography (CTPA) demonstrating multiple segmental pulmonary emboli in the right pulmonary artery (white arrows) at the time of the first CIP recurrence (August 2022).

Two months after the initial onset of CIP, the patient developed recurrent dyspnea on the sixth day of treatment with prednisone at a dosage of 30 mg/day, with radiological progression of pulmonary lesions (Figure 1E). High-dose methylprednisolone at 160 mg/day was reinstituted; after 7 days, the dosage was reduced to 80 mg/day for an additional 7 days, during which time both symptoms and imaging findings continued to improve (Figure 1F). During hospitalization, crackles and interstitial changes were noted, prompting the administration of pirfenidone (400 mg three times daily) because of its antifibrotic effects resulting from the inhibition of transforming growth factor-beta (TGF-β). Prednisone was prescribed at a dosage of 50 mg/day at discharge, and the dosage was tapered by 5 mg per week.

Two months after discharge, in November 2022, CT demonstrated significant resolution of pulmonary inflammation (Figure 1G), and prednisone was successfully discontinued, with a cumulative dosage equivalent to approximately 7,300 mg. The cumulative prednisone-equivalent dose was calculated item by item as follows: (1) 40 mg/day methylprednisolone × 7 days = 280 mg total methylprednisolone (equivalent to 350 mg prednisone); (2) before exacerbation at the first recurrence: 40 mg/day methylprednisolone × 7 days = 280 mg total methylprednisolone (equivalent to 350 mg prednisone); (3) 160 mg/day methylprednisolone × 9 days (1,440 mg total methylprednisolone, equivalent to 1,800 mg prednisone) followed by 80 mg/day methylprednisolone × 3 days (240 mg total methylprednisolone, equivalent to 300 mg prednisone); (4) oral prednisone before the second recurrence: 40 mg/day × 7 days plus 30 mg/day × 6 days = 460 mg total prednisone; (5) after the second recurrence: 160 mg/day methylprednisolone × 7 days (1,120 mg total methylprednisolone, equivalent to 1,400 mg prednisone) followed by 80 mg/day methylprednisolone × 7 days (560 mg total methylprednisolone, equivalent to 700 mg prednisone); and (6) after discharge: 50 mg/day oral prednisone, and the dosage was tapered by 5 mg weekly, corticosteroids were discontinued when the dose was to be reduced to 5 mg/day = 1,890 mg total prednisone. In summary, the total cumulative prednisone-equivalent dose was 7,250 mg, approximately 7,300 mg. Five months later (February 2023), the interstitial changes had largely resolved (Figure 1H), and pirfenidone was discontinued. Since then, CIP has not recurred. CIP was diagnosed clinically and radiologically, without histopathological evidence from lung biopsy; treatment response and resolution of interstitial pulmonary changes were assessed by serial chest CT.

### 2.3 Oncological follow-up and cerebral oligoprogression

After CIP had completely resolved, the patient did not receive any additional systemic antitumor therapy.

At 10 months after the onset of CIP, a comprehensive restaging evaluation demonstrated complete remission (CR) of the cervical lymph node and adrenal metastases, with stable pulmonary lesions.

At 14 months after the onset of CIP, a solitary left cerebellar metastasis measuring 1.7 × 1.6 cm was first detected (Figure 3A). Stereotactic body radiotherapy (SBRT) at a dose of 52 Gy in 13 fractions was given, and the lesion was reduced in size to 1.1 × 1.0 cm (Figure 3B).

**FIGURE 3:**
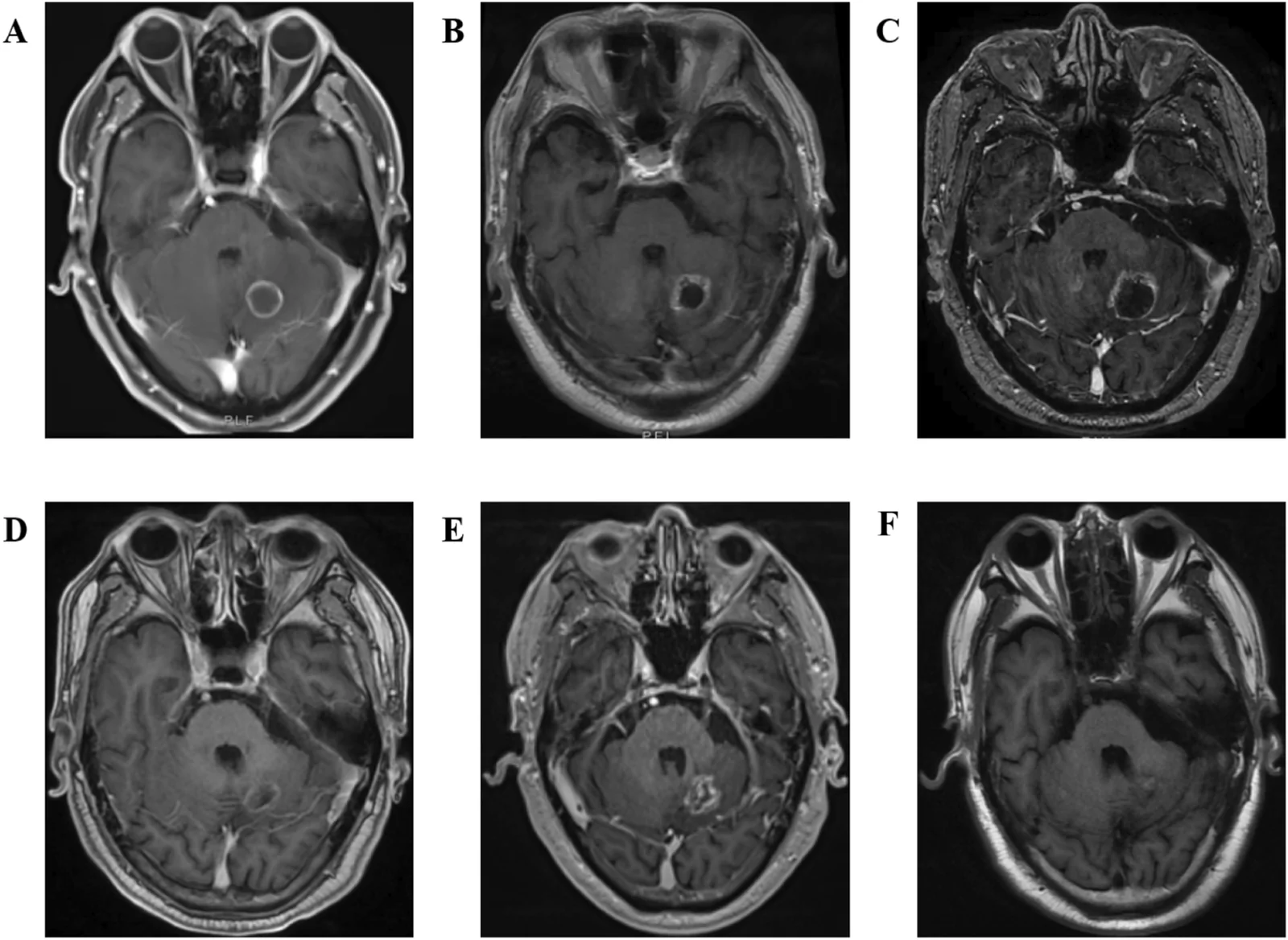
Sequential brain MRI during cerebral oligoprogression. **(A)** At 14 months after CIP onset (September 2023), initial detection of a solitary left cerebellar lesion measuring 1.7 × 1.6 cm. **(B)** After the first course of stereotactic body radiotherapy (SBRT, 52 Gy in 13 fractions) (November 2023), showing a reduction in size to 1.1 × 1.0 cm. **(C)** At 26 months after CIP onset (September 2024), showing the second instance of progression with ring enhancement and perilesional edema, with enlargement to 2.5 × 2.1 cm. **(D)** After four cycles of carboplatin, pemetrexed, and bevacizumab (November 2024), showing shrinkage to 1.6 × 1.2 cm and decreased edema size. **(E)** At 39 months after CIP onset (October 2025), showing the third instance of progression with enlargement to 2.0 × 1.6 cm. **(F)** After the second course of intensity-modulated radiotherapy (IMRT, 60 Gy in 25 fractions) (March 2026), showing a reduction in size to 1.9 × 1.3 cm.

At 26 months after the onset of CIP, the same solitary lesion had enlarged to 2.5 × 2.1 cm with associated edema (Figure 3C). As the patient declined a second course of radiotherapy for financial reasons, he received four cycles of carboplatin, pemetrexed, and bevacizumab, achieving good disease control, with the lesion being reduced in size to 1.6 × 1.2 cm and decreased edema (Figure 3D). However, bevacizumab was discontinued after the third and fourth cycles because of epistaxis and hemoptysis, which are known toxicities associated with VEGF inhibition. Carboplatin was discontinued following grade 3 myelosuppression after each of the second through fourth cycles; subsequently, during 6 months of pemetrexed maintenance monotherapy, recurrent grade 3 myelosuppression led to the discontinuation of all treatment regimens.

At 39 months after the onset of CIP, the cerebellar lesion increased in size again, measuring 2.0 × 1.6 cm (Figure 3E). Given the 25-month interval since the initiation of radiotherapy and acceptable normal tissue tolerance, a second course of intensity-modulated radiotherapy (IMRT) was administered at a dose of 60 Gy in 25 fractions, achieving good local control, with the lesion being reduced in size to 1.9 × 1.3 cm (Figure 3F). Despite this favorable local control, the nature of the cerebellar lesion remains uncertain. Notably, the dynamic changes in the cerebellar lesion were evaluated entirely by serial brain MRI. Because stereotactic biopsy of the cerebellar lesion carries a high procedural risk, it was not performed. Therefore, after multiple courses of brain radiotherapy, we could not definitively distinguish true tumor oligoprogression from radiation-induced necrosis or a mixture of the two. In addition, bevacizumab can result in radiological improvement in both tumor progression and radiation-induced necrosis, further complicating the interpretation of treatment response in this clinical setting.

### 2.4 Long-term drug toxicity: femoral head necrosis

At 34 months after the onset of CIP, the patient presented with right hip pain. MRI revealed bilateral femoral head necrosis (ONFH; ARCO stage III on the right side due to slight collapse and deformity, and stage I on the left side) (Figures 4A,B), and conservative management with celecoxib and weight-bearing restriction was initiated. By 46 months after the onset of CIP, the pain had worsened, and follow-up MRI demonstrated persistent progression of the right-sided lesion (ARCO stage III) (Figures 4C,D). At 48 months after the onset of CIP (the last follow-up), hip CT further confirmed advanced osseous changes of the right femoral head, including flattening, cystic and sclerotic changes, and joint space narrowing, consistent with advanced ONFH. Given that both the left middle cerebral artery and the internal carotid artery were stenosed and that consultation with the anesthesiologist indicated a high risk of perioperative cerebral infarction, the patient declined surgical intervention and opted to continue conservative management.

**FIGURE 4:**
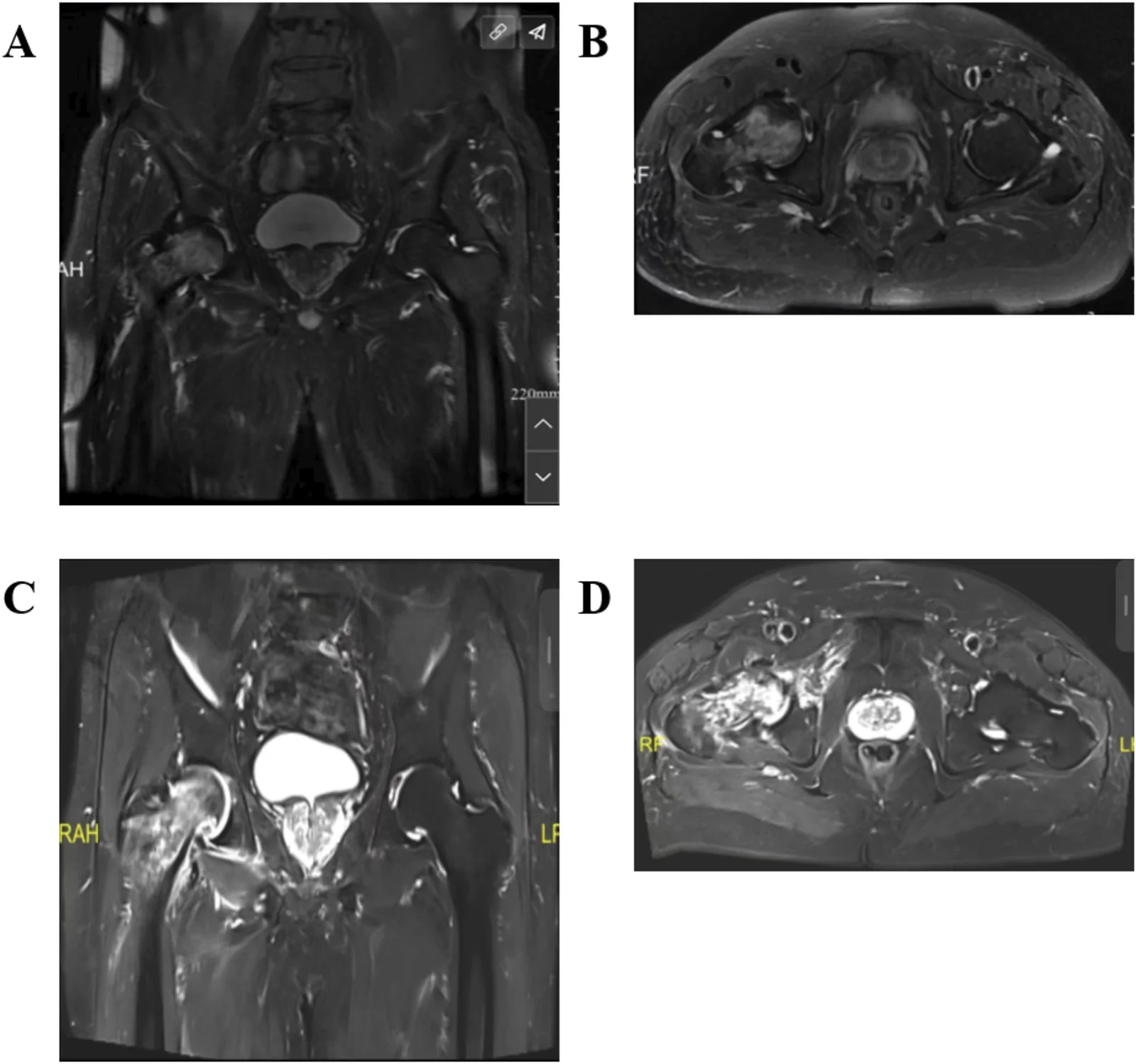
Bilateral hip MRI. **(A,B)** At 34 months after CIP onset (June 2025), showing bilateral femoral head osteonecrosis (ARCO stage III on the right side with slight collapse and deformity, and stage I on the left side), with more pronounced involvement on the right side. **(C,D)** At 46 months after CIP onset (March 2026), showing persistent progression of the right femoral head lesion (ARCO stage III).

As of June 2026, overall survival (OS) has exceeded 4 years, with an ECOG PS of 2, stable extracranial disease, and well-controlled brain metastases. Finally, it should be noted that ONFH was diagnosed by MRI according to the ARCO staging system; bone biopsy was not performed, and histopathological confirmation was therefore not possible.

### 2.5 Key clinical timeline

**Table:** 

Time point	Event	Key management	Outcome
April 2022	Diagnosis of stage IV lung adenocarcinoma	Sintilimab + chemotherapy × 4 cycles	PR
July 2022	Initial onset of CIP	40 mg/day methylprednisolone × 7 days	Improved
August 2022	First CIP recurrence + pulmonary embolism	160→80 mg/day methylprednisolone	Improved
September 2022	Second CIP recurrence	160→80 mg/day methylprednisolone + pirfenidone	CIP controlled
November 2022	Significant radiological resolution of CIP	Ultra-slow GC tapering and discontinuation	CIP resolution
February 2023	Interstitial changes largely resolved	Pirfenidone discontinuation	No CIP recurrence
May 2023	First comprehensive restaging	No systemic antitumor therapy administered	CR
September 2023	First cerebral oligoprogression	52Gy SBRT	Lesion shrinkage
September 2024	Second cerebral oligoprogression	Chemotherapy + bevacizumab × 4 cycles	Lesion shrinkage
June 2025	Right ONFH (stage III), left stage I	Conservative management	Pain
October 2025	Third cerebral oligoprogression	Second round of 60 Gy IMRT	Lesion shrinkage
March 2026	Right-sided ONFH progression (stage III)	Conservative management	Worsening pain
June 2026	Right ONFH, CT showing advanced osseous changes	Continued conservative management	OS > 4 years

## 3 Discussion

In the clinical management of severe immune-related toxicities, balancing aggressive anti-inflammatory intervention with long-term oncological surveillance represents a major pharmacological challenge. In the present case, both recurrences of CIP were directly attributable to rapid corticosteroid tapering. The ultimately successful regimen, consisting of 50 mg/day prednisone, with the dose being initially tapered by 5 mg per week, was considerably more conservative than the 4- to 6-week tapering schedule generally recommended by guidelines such as those from the National Comprehensive Cancer Network (NCCN) (Chinese Thoracic Society, Chinese Medical Association, Oncology Respiratory Disease Committee of China Anti-Cancer Association, Beijing Cancer Prevention and Treatment Research Association, 2025; Thompson et al., 2024). From a pharmacodynamic and pharmacokinetic perspective, the standard 6-week tapering regimen is insufficient to suppress inflammatory rebound in up to 30%–40% of patients with refractory CIP (Karayama et al., 2023; Karayama et al., 2024). Mechanistically, the abrupt discontinuation of exogenous corticosteroids triggers immediate T-cell activation and a surge of downstream cytokines—predominantly interleukin-6 (IL-6) and tumor necrosis factor-alpha (TNF-α)—before endogenous cortisol secretion via the hypothalamic–pituitary–adrenal axis is restored. The ultralow tapering strategy applied in this case provides sustained, low-level glucocorticoid receptor (GR) saturation, thereby preventing the reactivation of tissue-resident memory T cells while allowing physiological resetting of the HPA axis. (Arshad et al., 2024).

The clinical transition from acute alveolitis to fibrotic interstitial changes, heralded by Velcro crackles, signals disease progression into the fibrotic phase. Pirfenidone, an orally active small-molecule antifibrotic agent, exerts its effects primarily by downregulating TGF-β expression and inhibiting the TGF-β/Smad signaling pathway, thereby blocking the fibrotic cascade and synergistically suppressing fibroblast proliferation, myofibroblast differentiation, and extracellular matrix collagen deposition (Kudterkar and Wairkar, 2026). In severe CIP, the prolonged inflammatory phase frequently cross-activates fibrotic pathways, establishing a self-sustaining cycle of structural remodeling. Although large prospective clinical trials evaluating the use of antifibrotic agents in patients with CIP are limited, retrospective real-world data suggest that early dual therapy with corticosteroids and pirfenidone can successfully prevent irreversible distortion of the pulmonary parenchyma and reduce the likelihood of corticosteroid-refractory recurrence (Li et al., 2024). The optimal timing for initiating antifibrotic therapy in patients with CIP remains uncertain. Clinically, CIP can be broadly divided into an early acute alveolitis phase, predominantly characterized by inflammatory cell infiltration and ground-glass opacities, and a later fibrotic phase, which may be characterized by progressive interstitial remodeling, traction bronchiectasis, and Velcro crackles. For acute-phase CIP that responds rapidly to corticosteroids and is not complicated by major comorbidities, antifibrotic agents are generally not recommended as routine therapy (Chinese Thoracic Society, Chinese Medical Association, Oncology Respiratory Disease Committee of China Anti-Cancer Association, Beijing Cancer Prevention and Treatment Research Association, 2025; Thompson et al., 2024). In this patient, despite repeated high-dose corticosteroid therapy, inflammation recurred. Velcro crackles were audible on physical examination, which may suggest progression toward fibrotic interstitial injury. Therefore, pirfenidone was administered before fully irreversible fibrosis became evident on imaging, with the aim of suppressing the TGF-β-driven fibrotic cascade and potentially preventing permanent structural damage to the pulmonary parenchyma (Kudterkar and Wairkar, 2026; Li et al., 2024). This case suggests that antifibrotic therapy might be considered when clinical clues raise concern for early fibrotic transition, even in the absence of definitive radiological evidence of fibrosis. Prospective evidence remains limited, and such decisions should be individualized.

One particularly noteworthy aspect of this case is the pharmacological dissociation between highly durable 4-year extracranial remission and recurrent, isolated central nervous system (CNS) oligoprogression, following only brief exposure to sintilimab (four cycles). This clinical presentation provides *in vivo* evidence supporting the hypothesis of CNS immune privilege and sanctuary sites in barrier pharmacology. Sintilimab is a high-affinity humanized IgG4 monoclonal antibody targeting the PD-1 receptor. Structurally, its large molecular weight (approximately 150 kDa) limits its ability to cross the intact blood–brain barrier (BBB), with central-to-peripheral pharmacokinetic ratios typically falling below 0.1%–1.0% (Li et al., 2025). Consequently, although peripheral tissue-resident memory T cells undergo potent and durable reinvigoration following PD-1 blockade—maintaining rigorous systemic tumor surveillance without further drug administration—the CNS microenvironment remains a pharmacological sanctuary (Chuang et al., 2025; Gemelli et al., 2026; Kobeissy and Salzet, 2026). This sanctuary status is further reinforced by the unique neuroimmune microenvironment of the brain, characterized by a rich abundance of immunosuppressive factors (such as IL-10 and TGF-β) and local upregulation of PD-L1 expression on microglia and astrocytes, which collectively create a high threshold for the homing and functional execution of peripheral T-cell effectors (Andersen et al., 2021).

The pharmacological management of cerebellar progression further highlights the need for multitargeted, toxicity-driven regimen adjustments. Bevacizumab exerted both an antitumor antiangiogenic effect through VEGF pathway inhibition and a symptomatic benefit by reducing peritumoral edema via decreased microvascular permeability, enabling both clinical and radiological improvement even when the distinction between tumor progression and radiation necrosis remained challenging (Rata et al., 2026; Mittelman et al., 2025). However, its systemic antiangiogenic pharmacodynamics inevitably led to endothelial instability, manifesting as grade 2 epistaxis and hemoptysis, which resolved upon drug discontinuation. Concurrently, the patient developed severe myelosuppression driven by cumulative bone marrow toxicity caused by carboplatin and pemetrexed, illustrating the real-world challenge of maintaining treatment intensity when drug-induced toxicities intersect with long-term survival.

Finally, the development of bilateral ONFH at 34 months after the onset of CIP suggests a potential synergistic toxic mechanism between cumulative corticosteroid exposure and VEGF inhibition-mediated microvascular injury. The degree of cumulative corticosteroid exposure (approximately 7,300 mg prednisone equivalents) substantially exceeded the recognized nonlinear threshold of 4,000 mg, beyond which the risk of osteonecrosis accelerates exponentially (Krez et al., 2021; Muthu et al., 2023). Mechanistically, high-dose corticosteroids induce hypertrophy and hyperplasia of bone marrow adipocytes while simultaneously promoting osteoblast and osteocyte apoptosis, ultimately leading to intraosseous hypertension and focal compression of sinusoidal vessels, resulting in microcirculatory ischemia of the femoral head. Notably, the use of bevacizumab in this case may have resulted in a second synergistic toxic insult. By blocking VEGF-mediated signaling, bevacizumab severely impaired compensatory microvascular repair, angiogenesis, and osteogenic–angiogenic coupling, which are essential for the revascularization of ischemic bone tissue (Kakarala et al., 2025; Abebe et al., 2026). However, as this is a single-case observation, a direct causal synergistic effect of corticosteroids and bevacizumab in inducing ONFH cannot be confirmed. Further validation through large-scale clinical cohorts and animal experiments is required. Nevertheless, this potential molecular interplay warrants rigorous clinical vigilance.

Several limitations should be acknowledged. As a single case report, a causal relationship cannot be established. The absence of baseline quantitative PD-L1 scoring and TMB analysis limited our ability to map this durable peripheral response to specific genomic or immunobiological correlates. Furthermore, the lack of histopathological confirmation of cerebellar progression complicated the definitive distinction between true tumor recurrence and local radiation necrosis. Finally, although MRI-based diagnosis has high structural specificity for osteonecrosis of the femoral head, histopathological verification via bone biopsy was not possible. Nevertheless, the longitudinal follow-up of 48 months provides profound real-world translational insights into long-term drug safety and multi-regimen pharmacovigilance.

## 4 Conclusion

For patients with severe recurrent CIP, ultralow corticosteroid tapering combined with adjunctive pirfenidone represents an effective strategy for controlling inflammation. Highly durable peripheral immunological memory and extracranial remission may persist long after ICI discontinuation, even when the CNS serves as an isolated sanctuary site for oligoprogression. This presentation is consistent with the hypothesis that the brain is an immunologically privileged site. This case suggests a potential synergistic toxic mechanism between high-dose corticosteroids and anti-VEGF therapy in the development of late-onset musculoskeletal toxicity. Clinicians should remain vigilant for such toxicity and should commit to rigorous long-term pharmacovigilance for ICI survivors.

### 4.1 Patient perspective

“After developing pneumonitis, my doctor told me I had to stop immunotherapy, and I was terrified that the tumor would come back immediately. To my surprise, the lung and other lesions remained stable all along—only the brain lesion required repeated interventions. What bothers me most now is the hip pain, which affects both walking and sleeping. My doctor said surgery is too risky, so I have no choice but to continue conservative management. I'm grateful to have survived more than four years, and I hope my experience can offer some reference for other patients facing similar challenges.”

## Data Availability

The original contributions presented in the study are included in the article/supplementary material, further inquiries can be directed to the corresponding author.
